# Wort Substrate Consumption and Metabolite Production During Lambic Beer Fermentation and Maturation Explain the Successive Growth of Specific Bacterial and Yeast Species

**DOI:** 10.3389/fmicb.2018.02763

**Published:** 2018-11-19

**Authors:** Jonas De Roos, Peter Vandamme, Luc De Vuyst

**Affiliations:** ^1^Research Group of Industrial Microbiology and Food Biotechnology, Bioengineering Sciences Department, Vrije Universiteit Brussel, Brussels, Belgium; ^2^Laboratory for Microbiology, Department of Biochemistry and Microbiology, Ghent University, Ghent, Belgium

**Keywords:** lambic beer, *Dekkera*, malolactic fermentation, MALDI-TOF MS, amplicon sequencing

## Abstract

The present study combined high-throughput culture-dependent plating and culture-independent amplicon sequencing with a metabolite target analysis to systematically dissect the identity, evolution, and role of the microorganisms, substrates, and metabolites during the four-phase fermentation and maturation process of lambic beer production. This led to the following new insights. The changing physicochemical parameters and substrate and metabolite compositions of the fermenting wort and maturing lambic beer provoked several transitions between microbial species and explained the four-step production process. Manual wort acidification with lactic acid shortened the enterobacterial phase and thus kept biogenic amine formation by enterobacteria present during the early stages of fermentation at a minimum. Growth advantages during the alcoholic fermentation phase caused a transition from the prevalence by *Hanseniaspora uvarum* and *Kazachstania* species to that by *Saccharomyces cerevisiae* and later on *Saccharomyces kudriavzevii*, due to changing environmental parameters. During the acidification phase, *Pediococcus damnosus* was prevalent and performed a malolactic fermentation. *Acetobacter pasteurianus* produced acetic acid and acetoin. Upon maturation, *Dekkera* species appeared, together with *P. damnosus* and *Pichia membranifaciens*, thereby contributing to acetic acid production, depending on the oxygen availability. Moreover, the *Dekkera* species consumed the acetoin produced by the acetic acid bacteria for redox balancing. The breakdown of maltooligosaccharides seemed to be independent of the occurrence of *Dekkera* species and started already early in the fermentation process.

## Introduction

Worldwide, the beer market primarily consists of lager beers produced through bottom fermentation of barley malt, water, and hop with the brewers’ yeasts *Saccharomyces bayanus* or *Saccharomyces pastorianus* (Bokulich and Bamforth, 2013, 2017; Howard, 2014). However, top-fermented ale beers now know high production volumes as well, either on a global scale or regionally. Regional production often takes place according to traditional recipes that make use of a variety of ingredients (e.g., diverse cereals, herbs, and spices). Top-fermented beer production is carried out mainly with *Saccharomyces cerevisiae* (Briggs et al., 2004). In contrast, autochthonous microorganisms [spontaneously growing yeasts and lactic acid bacteria (LAB)] or uncharacterized mixed starter cultures (yeast-LAB starter cultures maintained in-house) perform the fermentation and/or maturation processes of certain traditional Belgian acidic ales (Verachtert and Iserentant, 1995; Martens et al., 1997; Verachtert and Derdelinckx, 2005; Snauwaert et al., 2016). Belgian lambic beers are a typical example of spontaneously fermented acidic beers, which are produced in specialized breweries (Spitaels et al., 2014, 2015, 2017). Traditional acidic beers are currently attracting increasing attention worldwide (Pothakos et al., 2016; Spitaels et al., 2017; De Roos and De Vuyst, 2018). American craft brewers for example have adopted the production methods of Belgian-style acidic ales to produce spontaneously fermented American coolship ales (ACAs; Bokulich et al., 2012).

Acidic lambic beers are obtained through spontaneous fermentation of barley malt, unmalted wheat, and aged dry hops (De Keersmaecker, 1996; Pothakos et al., 2016; Spitaels et al., 2017). The spontaneous fermentation and maturation process can proceed up to 3 years and is traditionally carried out in wooden casks (Verachtert and Iserentant, 1995). Most knowledge about these beers originates from microbiological studies performed several decades ago. These studies relied entirely on culture-dependent microbiological analyses and a restricted metabolite target analysis with techniques that are outdated now (Van Oevelen et al., 1976, 1977; Spaepen et al., 1978, 1979; Verachtert, 1983; Verachtert and Dawoud, 1984; Verachtert et al., 1989; Martens et al., 1991, 1992; Shanta Kumara and Verachtert, 1991; Verachtert and Iserentant, 1995). Most of these studies have shown that four phases can be distinguished during a traditional lambic beer production process: (i) the enterobacterial and wild (oxidative) yeast phase from the start up to 1 month of fermentation, which is characterized by low concentrations of organic acids and ethanol; (ii) the main fermentation phase (from month 1 until month 4) dominated by the yeast species *S. cerevisiae* and *S. pastorianus*, which is characterized by the production of ethanol and carbon dioxide; (iii) the acidification phase (from month 4 until month 10) dominated by the LAB species *Pediococcus damnosus* and/or *Lactobacillus brevis*, which is characterized by the production of high concentrations of lactic acid; and (iv) the maturation phase (after 10 months) dominated by the yeast species *Dekkera bruxellensis* and the LAB species *P. damnosus* and/or *L. brevis*, which is characterized by the production of ethyl acetate and ethyl lactate (Verachtert and Iserentant, 1995; Verachtert and Derdelinckx, 2005).

Recently, lambic beer production processes performed in a traditional and common brewery were revisited through a follow-up for 24 and 12 months, respectively, applying primarily up-to-date culture-dependent microbiological analysis on samples taken as a function of time but without monitoring substrate consumption and metabolite production (Spitaels et al., 2014, 2015). These studies have shown the same succession of microbial species mentioned above, albeit that manual acidification of the wort with lactic acid before the start of the fermentation at the common brewery, which is an adapted practice in most today’s lambic breweries too, eliminates the first enterobacterial phase and results in an overlap of the acidification and maturation phases. Acidification would avoid biogenic amine production by enterobacteria (Spitaels et al., 2015). Yet, the effect on the total biogenic amine contents of these beers is unclear, as not only enterobacteria but also other lambic beer inhabitants such as LAB species and *D. bruxellensis* are able to produce these compounds (Izquierdo-Pulido et al., 1995, 1996; Kalac and Krizek, 2003; Oelofse et al., 2008; Spano et al., 2010). Further, it has been shown that acetic acid bacteria (AAB) are present abundantly during major periods of the first fermentation year of traditional lambic beer production and produce significantly higher concentrations of acetic acid and acetoin at the liquid/air interphase of the casks, due to higher AAB counts and metabolic activity compared to the middle and bottom of the fermenting wort and maturing beer in the casks (De Roos et al., 2018). ACA production closely parallels the microbial succession seen during lambic beer production. Minor differences between both beer productions occur in the enterobacterial species present and the abundant presence of *S. cerevisiae* from the start of ACA production, the latter maybe due to the production of ACAs in a common brewery. Further, aerobic bacteria, including AAB, become more prevalent after 1 year of ACA production, although *Lactobacillales* remain dominant (Bokulich et al., 2012). This contrasts with certain lambic beer production processes, in which AAB do occur until 24 months of maturation, but are most prevalent after 3 months of fermentation (De Roos et al., 2018). Hence, it is not yet clear how shaping of the microbial communities during lambic beer fermentation and maturation occurs.

The present study aimed at a systematic dissection of the identity, evolution, and role of the microorganisms, substrates, and metabolites in oak casks used for the spontaneous fermentation and maturation process of Belgian, traditional lambic beer. Therefore, high-throughput culture-dependent and culture-independent microbiological techniques, in combination with a metabolite target analysis of fermentation- and maturation-related compounds, were performed during a production period of 24 months.

## Materials and Methods

### Lambic Beer Production and Sampling

Two lambic beer production processes were sampled in a traditional lambic brewery located in the Senne river valley southwest of Brussels (Belgium) in November 2014; these were the same processes, for which the AAB communities were studied, as reported before (De Roos et al., 2018). Briefly, wort of 12.6°P was prepared according to the brewer’s recipe, manually acidified with lactic acid after wort boiling, and cooled overnight in a coolship open to the environmental air. The wort was then chilled to 4°C and transferred by means of appropriate tubings into oak casks of 660 l for fermentation and maturation. These casks originated from port wine production but were already used several times to produce lambic beer. Before use, the casks were cleaned with high-pressurized water and fumigated with sulfur sticks to inhibit mold growth after cleaning. Before filling, the casks were opened to the environmental air to remove the remaining sulfur vapors. Two identical casks (further referred to as casks 1 and 2), filled with wort of the brew mentioned above and located next to each other in the bottom row of tens of casks in a cellar (ambient temperature ranging from 9 to 20°C, due to seasonal effects), were sampled as a function of time, representing biological duplicates.

A sample of 100 ml was taken from the cooled wort before its transfer to the wooden casks. Further samples (100 ml) were taken 1 h; 1 and 3 days; 1, 2, 3, and 7 weeks; and 3, 6, 7.5 (only for LAB in cask 2), 9, 13, 18, and 24 months after its transfer. A volumetric adjustment of the liquid level in the casks was only performed at the timepoint of 13 months Aseptic sampling was performed at different heights of the fermenting wort and maturing beer in the casks (top, middle, and bottom) by applying flame sterilization and making use of γ-irradiated jumbo pipets (900 mm, high-density polyethylene; VWR International, Darmstadt, Germany) through the cork-plugged bunghole of the casks (50 ml per insertion). Samples were put on ice before their transfer to the laboratory for analysis. Part of the samples was analyzed immediately (physicochemical parameters and microbiological plating); another part (50 ml) was centrifuged (4618 × *g*, 20 min, 4°C) to obtain cell pellets and cell-free supernatants that were stored at -20°C for microbiological and metabolite target analysis, respectively.

### Determination of Physicochemical Parameters

Immediately after sampling, the pH, temperature, and apparent attenuation were measured on site. The pH was determined with a portable pH meter (pH10 Pen; VWR International). Temperature and apparent attenuation were determined with a portable density meter (DMA 35; Anton Paar, Graz, Austria).

### Culture-Dependent Microbiological Analyses

#### Selective Plating and Culturing

The chilled samples were serially diluted in saline (0.85%, m/v, NaCl) and 100 μl of each dilution was plated onto three selective agar media, which were chosen based on previous research (Spitaels et al., 2014, 2015): (i) modified de Man-Rogosa-Sharpe (mMRS) agar medium for the enumeration and isolation of presumptive LAB after anaerobic incubation (AnaeroGen; Thermo Fisher Scientific, Waltham, MA, United States) at 20°C for 10 days to inhibit the growth of AAB; mMRS agar medium consisted of MRS agar (Oxoid, Basingstoke, Hampshire, United Kingdom) supplemented with a 0.2 ppm vitamin solution (cobalamin, folic acid, nicotinic acid, pantothenic acid, pyridoxal phosphate, and thiamine) to enhance the chance to isolate nutritionally depending LAB species, and with 5 ppm of amphotericin B (Sigma-Aldrich, Bornem, Belgium) and 200 ppm of cycloheximide (Sigma-Aldrich) to inhibit fungal growth; (ii) yeast extract-peptone-glucose (YPG) agar medium [2.0% glucose (Merck, Darmstadt, Germany), 0.5% yeast extract (Merck), 1.0% peptone (Oxoid), and 1.5% agar (Oxoid); m/v] for the enumeration and isolation of presumptive yeasts after aerobic incubation at 30°C for 7 days; YPG medium was supplemented with 200 ppm of chloramphenicol to inhibit bacterial growth; and (iii) YPGc agar medium, i.e., YPG agar medium supplemented with 50 ppm of cycloheximide to select for presumptive cycloheximide-resistant yeasts after aerobic incubation at 30°C for 7 days.

#### Enumeration and Isolation of Colonies

Agar media containing 30 to 300 colony forming units (CFU) were counted to determine the microbial community dynamics. In the case of AAB, the average counts obtained on modified deoxycholate-mannitol-sorbitol (mDMS) agar medium for samples withdrawn from the same fermenting wort and maturing beer in the casks of the same lambic beer production processes but reported before were taken (De Roos et al., 2018). Subsequently, 10% of the total number of colonies from appropriate dilutions of the respective agar media were randomly picked to determine the culture-dependent microbial species diversity.

#### Dereplication and Identification of Microbial Isolates by MALDI-TOF MS

For dereplication and identification of the presumptive yeast and LAB isolates, each colony was sub-cultivated twice on its respective agar medium. The resulting third-generation colonies were used for matrix-assisted laser desorption ionization-time of flight mass spectrometry (MALDI-TOF MS) fingerprinting and mass spectra processing, as described previously for AAB (De Roos et al., 2018). Representative strains from each cluster, obtained through numerical analysis of the mass spectral profiles, were finally identified through comparative sequence analysis of the 16S rRNA gene for bacterial isolates and the internal transcribed spacer (ITS) region for yeast isolates, as described previously, and were considered identified to species level when the identity was higher than 97% (Spitaels et al., 2014). The identities of the closest known relatives (type strains) were obtained using the basic local alignment search tool (BLAST) algorithm and by comparison with the GenBank database of the National Center for Biotechnology Information (NCBI, Bethesda, MD, United States^1^; Altschul et al., 1990). In the case AAB identities were needed, they were taken from the data on the AAB published previously (De Roos et al., 2018).

### Culture-Independent Microbiological Analyses

Culture-independent analysis was performed on fermenting wort and maturing beer samples from cask 1 to check if some microbial groups were missed throughout sampling of the lambic beer production processes, due to culture-dependent biases such as the appearance of microorganisms in a viable but not culturable state and/or the possibility that the isolation agar media used favored the cultivation of specific microorganisms.

#### Total DNA Extraction

To enable culture-independent microbial species diversity analysis, total DNA extracts were prepared from the cell pellets obtained from the original samples, following a method combining enzymatic, chemical, and mechanical treatments for cell lysis and phenol/chloroform/isoamyl alcohol treatment and column purification to extract and purify the DNA, as described before (Vermote et al., 2018).

#### Amplification of Group-Specific Loci

Amplification of group-specific loci was performed as described previously (De Bruyn et al., 2017). Therefore, specific loci of bacterial and fungal marker genes were amplified with primers purchased from Integrated DNA Technologies (Leuven, Belgium) through PCR. The V4 hypervariable region of the bacterial 16S rRNA gene was amplified with the primers F515 (5′-TCGTCGGCAGCGTCAGATGTGTATAAGAGACAGGTGTGCCAGCMGCCGCGGTAA-3′) and R806 (5′-GTCTCGTGGGCTCGGAGATGTGTATAAGAGACAGG GACTACHVGGG TWTCTAAT-3′) (Caporaso et al., 2011), both with a specific Illumina platform 5′ tag (underlined). The thermal PCR program was run under the following conditions: denaturation at 94°C for 3 min, followed by 35 amplification cycles at 94°C for 45 s (denaturation), 50°C for 60 s (annealing), and 72°C for 90 s (extension), and final elongation at 72°C for 10 min. The fungal ITS1 region of the rDNA was amplified with the primers BITS (5′-TCGTCGGCAGCGTCAGATGTGTATAAGAGACAGACCT GCGGARGGATCA-3′) and B58S3 (5′-GTCTCGTGGGCTC GGAGATGTGTATAAGAGACAGGAGATCCRTTGYTRAAAG TT-3′) (Bokulich and Mills, 2013), both with a specific Illumina platform 5′ tag (underlined). The thermal PCR program was run under the following conditions: denaturation at 95°C for 2 min, followed by 40 amplification cycles at 95°C for 30 s (denaturation), 50°C for 30 s (annealing), and 72°C for 60 s (extension), and final extension at 72°C for 5 min. A commercially available bacterial mock community (HM-782D; BEI Resources, Manassas, VA, United States) was amplified simultaneously with all samples to detect sequencing errors afterward. PCR sample mixtures were prepared and checked for the appropriate length, as described before (De Bruyn et al., 2017).

#### High-Throughput Sequencing of the V4 and ITS1 Amplicons

High-throughput sequencing of the V4 and ITS1 amplicons was performed, as described previously (De Bruyn et al., 2017). Therefore, the PCR amplicons obtained were purified using the Wizard SV gel and PCR clean-up system (Promega, Madison, WI, United States), following the manufacturer’s instructions, after which they were eluted into 30 μl of nuclease-free water. Subsequently, they were subjected to size selection, using Agencourt AMPure XP PCR purification magnetic beads (Beckman Coulter, Brea, CA, United States), according to the manufacturer’s instructions, except that the relative amount of bead solution was changed to 1.1× for the BITS/B58S3 primer set, Eppendorf tubes were left to dry to the open air for maximally 3 min, and elution was done into 30 μl of nuclease-free water. The quality and concentrations of the amplicons were validated, as described before (De Bruyn et al., 2017). Finally, the V4 and ITS1 amplicons originating from the same sample were pooled in a 2:1 molar ratio and barcoded with the same index prior to sequencing. All samples were paired-end sequenced together with the amplified bacterial mock community, using the Illumina MiSeq platform (Illumina, San Diego, CA, United States) at the interuniversity VUB-ULB sequencing facility (BRIGHTcore, Jette, Belgium). Two Fastq files were obtained for each sample, containing all forward and reverse reads from both bacterial and fungal amplicons.

#### Bioinformatics Analysis

The two Fastq files of each sample (forward and reverse sequences), containing both bacterial V4 and fungal ITS1 sequences, were split into an additional two files, containing only forward and reverse sequences of the V4 and ITS1 regions, respectively, as described previously (De Bruyn et al., 2017). Therefore, different workflows were followed to process the bacterial and fungal sequences. For the bacterial V4 sequences (291 base pairs) of all samples, including the mock community, primers were removed (reverse primer for the forward sequences and forward primer for the reverse sequences, respectively) using the Cutadapt software (Martin, 2011), before subjection to the mothur software version 1.36.1 (Schloss et al., 2009). After generation of the contigs, the unique sequences were clustered into groups based on a maximum of two mismatches. Chimeric sequences were removed with the UCHIME algorithm (Edgar et al., 2011). The most abundant sequence of each group was chosen as the representative one and was taxonomically assigned by alignment against the bacterial 16S rRNA SILVA database (release 132) to remove non-bacterial reads. Afterward, the representative unique sequences that were assigned the same “order” taxon were clustered together. Within each cluster, multiple alignment analysis was performed to group the sequences into operational taxonomic units (OTUs) at a level of 97% identity. The sequence error rate was estimated in mothur by comparing the sequenced mock community against the *in silico* mock community. Regarding the fungal ITS1 sequences, which could vary in length, the forward and reverse sequences were trimmed using the Cutadapt software to avoid possible adapter read-throughs of ITS1 sequences shorter than the 300-base pair chemistry used by the Illumina platform (Gweon et al., 2015). The trimmed files were then quality-screened and further processed using the mothur software to generate contigs, as described before (De Bruyn et al., 2017). In particular, the unique sequences were classified taxonomically through comparison with the fungal UNITE_ITS1 database (v6_sh_99), and merged into OTUs when the taxonomic allocation was identical (Findley et al., 2013).

### Substrate and Metabolite Target Analyses

Substrate and metabolite concentrations were determined in the cell-free supernatants obtained from the original samples. Quantification was performed through external calibration, unless stated otherwise. All samples were both prepared and analyzed in triplicate.

#### Determination of Simple Carbohydrate Concentrations

The concentrations of fructose, glucose, maltose, and sucrose were measured by high-performance anion exchange chromatography coupled to pulsed amperometric detection (HPAEC-PAD) with internal standardization, as described before (De Roos et al., 2018). Briefly, an ICS3000 chromatograph (Dionex, Sunnyvale, CA, United States) equipped with a Carbopac^TM^ PA10 column (Dionex) and coupled to a pulsed amperometric detector (Dionex) was used. The same mobile phase and eluent gradient were applied. All samples were deproteinized, vortexed, centrifuged (21,912 × *g*, 15 min, 4°C), and filtered (0.2-μm pore-size Whatman filters; GE Healthcare Life Sciences, Bucks, United Kingdom) before injection (10 μl) into the column.

#### Determination of Maltooligosaccharide Concentrations

The concentrations of maltotriose, maltotetraose, maltopentaose, maltohexaose, and maltoheptaose were measured by HPAEC-PAD with internal standardization, as described before for maltotriose (De Roos et al., 2018). Briefly, an ICS3000 chromatograph (Dionex) equipped with a Carbopac^TM^ PA100 column (Dionex) and coupled to a pulsed amperometric detector (Dionex) was used. The same mobile phase and eluent gradient were applied. All samples and standards were deproteinized, vortexed, centrifuged, and filtered, as described above, before injection (10 μl) into the column.

#### Determination of Ethanol and Short Chain Fatty Acid Concentrations

The concentrations of ethanol, acetic acid, propionic acid, and butyric acid were measured by high-performance liquid chromatography with refractive index detection (HPLC-RI), applying external calibration, as described before for ethanol and acetic acid (De Roos et al., 2018). Briefly, a Waters chromatograph (Waters, Milford, MA, United States) equipped with an ICSep ICE ORH-801 column (Transgenomic North America, Omaha, NE, United States) and coupled to a RI detector (Waters) was used. The same mobile phase and eluent flow rate were used. All samples and standards were deproteinized, vortexed, centrifuged, and filtered, as described above, before injection (30 μl) into the column.

#### Determination of Lactic Acid Stereoisomer Concentrations

The concentrations of D- and L-lactic acid were measured by HPLC with ultraviolet detection (HPLC-UV) and external calibration, based on a method described before, except that sample and standard preparations were deproteinized by adding 300 μl of Carrez A solution [36 g/l of K_4_Fe(CN)_6_.3H_2_O] and 300 μl of Carrez B solution (72 g/l of ZnSO_4_.7H_2_O) to 600 μl of analyte (Laureys and De Vuyst, 2014). They were analyzed with a Waters chromatograph equipped with a Shodex ORpak CRX-853 column (Showa Denko, Tokyo, Japan) and coupled to a UV detector operating at 253 nm (Waters). The mobile phase consisted of 10% acetonitrile (Fisher Chemical, Loughborough, United Kingdom) and 90% 1 mM CuSO_4_ (Merck) at a flow rate of 1 ml/min. All samples and standards were vortexed, centrifuged, and filtered, as described above, before injection (30 μl) into the column.

#### Determination of Volatile Concentrations

The concentrations of acetoin, 2,3-butanediol, 2,3-butanedione, ethyl acetate, ethyl lactate, isoamyl acetate, (iso)butyric acid, (iso)valeric acid, methyl-1-butanol, and propionic acid were measured by gas chromatography with flame ionization detection (GC-FID), applying internal standardization, as described before for ethyl acetate and acetoin (De Roos et al., 2018). Briefly, a Focus gas chromatograph (Interscience, Breda, Netherlands) equipped with a Stabilwax-DA column (Restek, Bellefonte, PA, United States) coupled to a FID-80 detector (Interscience) was used. All operating conditions were the same. All samples and standards were deproteinized, vortexed, centrifuged, and filtered as described above, before injection (1 μl; split 40) into the column.

#### Determination of Organic Acid Concentrations

The concentrations of citric acid, gluconic acid, malic acid, and succinic acid were measured by ultra-performance liquid chromatography with tandem mass spectrometric detection (UPLC-MS/MS), applying external calibration, as described before for gluconic acid (De Roos et al., 2018). Briefly, an Acquity system chromatograph (Waters) equipped with a HSS T3 column (Waters) and coupled to a TQ tandem mass spectrometer with a ZSpray^TM^ electrospray ionization source in negative ionization mode (Waters) was used. The same mobile phase and eluent gradient were applied. All samples and standards were deproteinized, vortexed, centrifuged, and filtered, as described above, before injection (10 μl) into the column.

#### Determination of Biogenic Amine Concentrations

The concentrations of agmatine, cadaverine, histamine, 2-phenylethylamine, putrescine, tryptamine, and tyramine were measured by UPLC-MS/MS, using multiple reaction monitoring and internal standardization. Preparation of samples and standards involved predilution in ultrapure water followed by deproteinization. For the latter, 600 μl of acetonitrile (Fisher Chemical) was added to 300 μl of analyte plus 300 μl of internal standard (N,N-dimethylcyclohexylamine; Fluka Chemie, Buchs, Switzerland). They were analyzed with an Acquity chromatograph equipped with a HSS T3 column coupled to a TQ tandem mass spectrometer with a ZSpray^TM^ electrospray ionization source used in positive ionization mode (Waters). The mobile phase consisted of 950 ml of ultrapure water, 50 ml of acetonitrile (Fisher Chemical), 10 mM ammonium acetate (Merck), and 2 ml of formic acid (Merck) (eluent A), and 50 ml of ultrapure water, 950 ml of acetonitrile, 10 mM ammonium acetate, and 2 ml of formic acid (eluent B), at a flow rate of 0.3 ml/min, with the following gradient: 0.0–1.0 min, isocratic 100% A; 1.0–1.6 min, linear from 100 to 90% A and from 0 to 10% B; 1.6–3.4 min, isocratic 90% A and 10% B; 3.4–3.5 min, linear from 90 to 40% A and from 10 to 60% B; 3.5–6.0 min, isocratic 40% A and 60% B; 6.0–6.2 min, linear from 40 to 100% A; and 6.2–7.5 min, isocratic 100% A. The following mass spectrometric settings were used: capillary voltage, 3.50 kV; cone voltage, 10–23 V, depending on the biogenic amine detected; source temperature, 120°C; desolvation temperature, 350°C; cone gas flow, 50 l/h; desolvation gas flow, 550 l/h; and collision energy, 11–15 eV, depending on the biogenic amine detected. All samples and standards were vortexed, centrifuged, and filtered, as described above, before injection (10 μl) into the column.

## Results

### Course of pH, Temperature, and Apparent Attenuation

**FIGURE 1:**
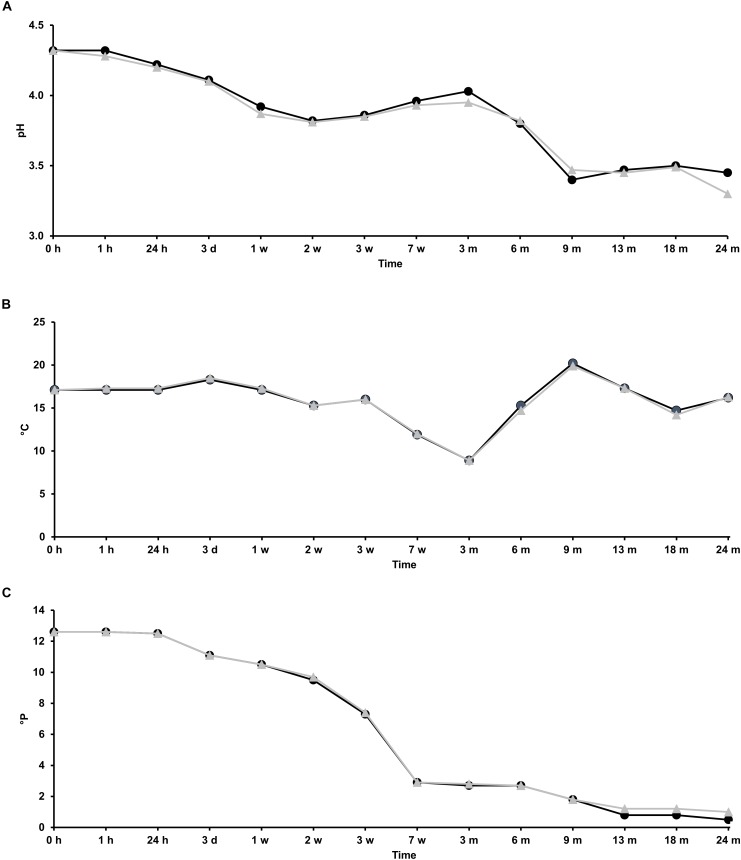
Course of the physicochemical parameters pH **(A)**, temperature **(B)**, and apparent attenuation **(C)** in the fermenting wort and maturing beer during the 24-month lambic beer production processes carried out in two identical oak casks. Cask 1 (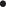) and cask 2 (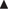).

**FIGURE 2:**
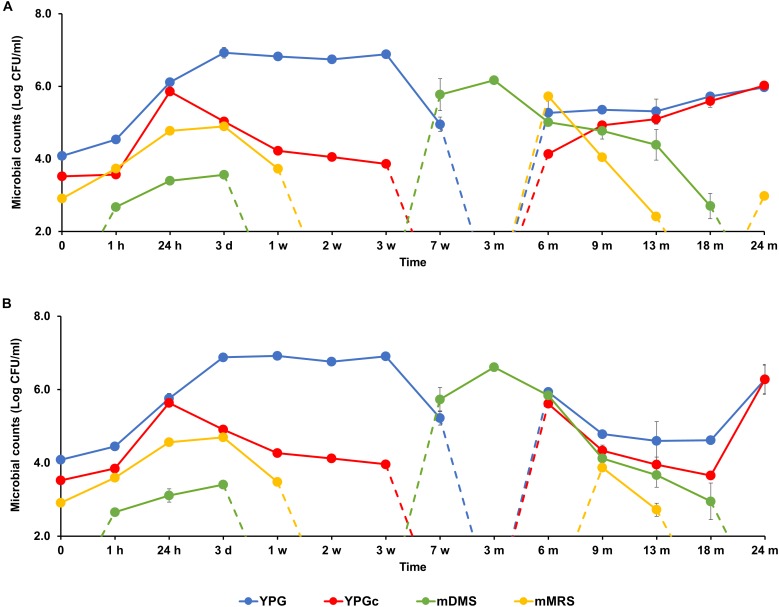
Counts of presumptive yeasts, cycloheximide-resistant yeasts, acetic acid bacteria (AAB), and lactic acid bacteria (LAB), as plated on yeast-peptone-glucose (YPG) agar medium, yeast-peptone-glucose agar medium supplemented with cycloheximide (YPGc), modified deoxycholate-mannitol-sorbitol (mDMS) agar medium, and modified de Man-Rogosa-Sharpe (mMRS) agar medium, respectively, during the 24-month lambic beer production processes carried out in two identical oak casks. Since no differences were found regarding yeast and LAB enumerations for the different sampling heights (top, middle, and bottom of the fermenting wort and maturing beer in the casks), average counts are reported. For the AAB, the average was taken of the counts from fermenting wort and maturing beer samples taken at the top, middle, and bottom of the same casks during the same lambic beer production processes reported before (De Roos et al., 2018). Cask 1 **(A)** and cask 2 **(B)**.

**FIGURE 3:**
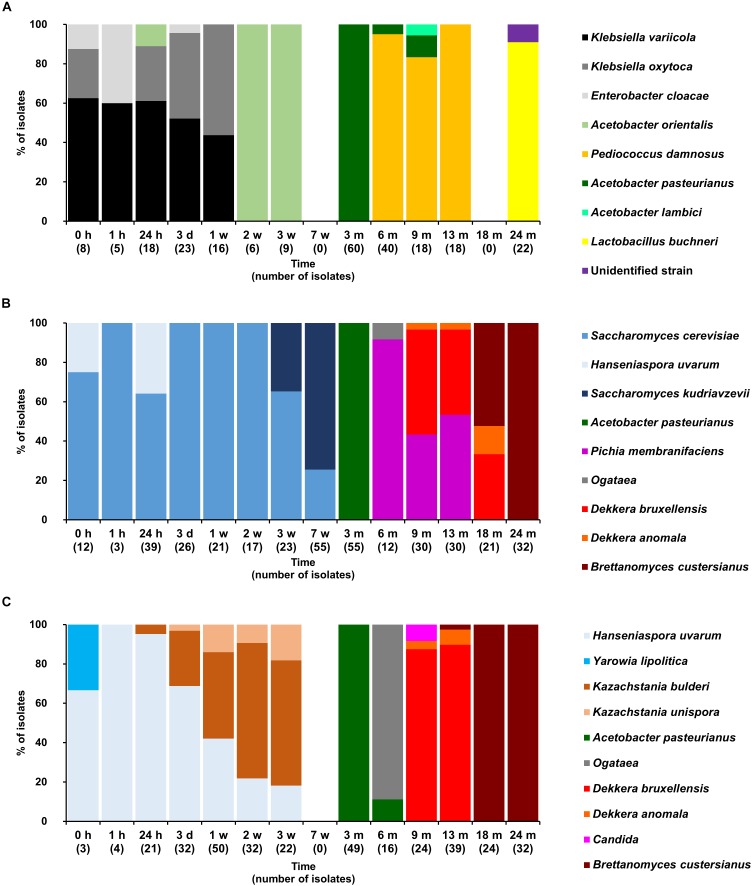
Community dynamics of the LAB **(A)**, yeast **(B)**, and cycloheximide-resistant yeast **(C)** species during the 24-month lambic beer production process carried out in cask 1. The following bacterial species were identified: *Klebsiella variicola* (GenBank accession number NR_025635.1), *Klebsiella oxytoca* (NR_112010.1), *Acetobacter orientalis* (NR_028625.1), *Enterobacter cloacae* (CP017184.1), *Pediococcus damnosus* (NR_042087.1), *Acetobacter pasteurianus* (CP015168.1), *Acetobacter lambici* (HG329531.1), and *Lactobacillus buchneri* (NR_041293.1). The following yeast species were identified: *Saccharomyces cerevisiae* (KC881067.1), *Hanseniaspora uvarum* (KY103558.1), *Saccharomyces kudriavzevii* (NR_111355.1), *Pichia membranifaciens* (NR_111195.1), *Ogataea* sp. (KY104429.1), *Dekkera bruxellensis* (KY103308.1), *Dekkera anomala* (KY103306.1), *Dekkera custersianus* (NR_138184.1). *Yarrowia lipolytica* (KY105973.1), *Kazachstania bulderi* (NR_138198.1), *Kazachstania unispora* (KY103682.1), and *Candida* sp. (NR_137645.1).

**FIGURE 4:**
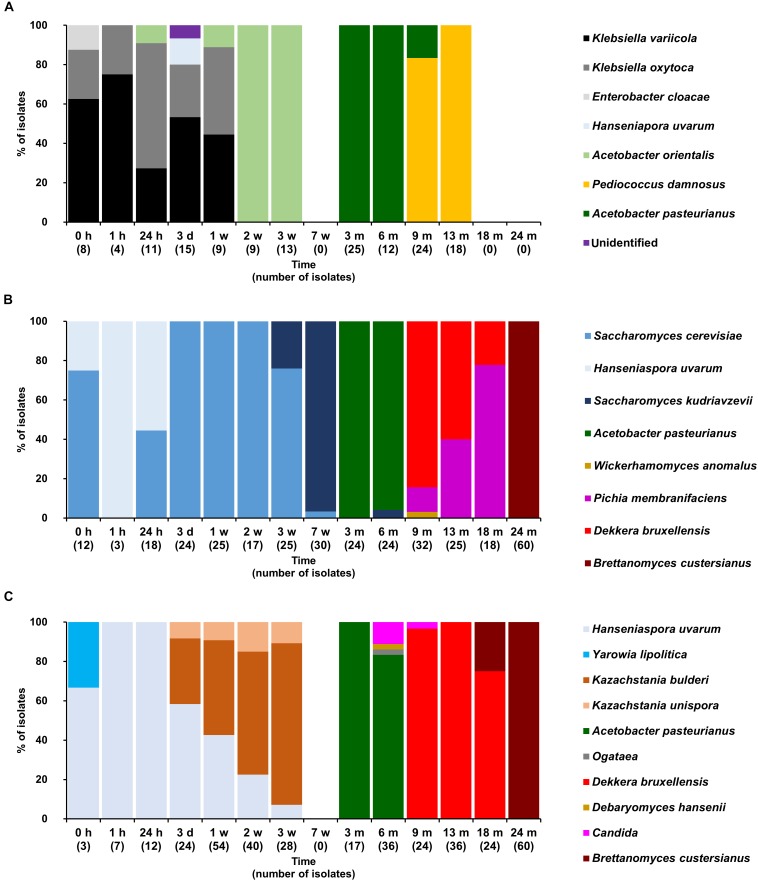
Community dynamics of the LAB **(A)**, yeast **(B)**, and cycloheximide-resistant yeast **(C)** species during the 24-month lambic beer production process carried out in cask 2. The following microbial species were identified: *Klebsiella variicola* (GenBank accession number NR_025635.1), *Klebsiella oxytoca* (NR_112010.1), *Enterobacter cloacae* (CP017184.1), *Hanseniaspora uvarum* (KY103558.1), *Acetobacter orientalis* (NR_028625.1), *Pediococcus damnosus* (NR_042087.1), *Acetobacter pasteurianus* (CP015168.1), *Saccharomyces cerevisiae* (KC881067.1), *Saccharomyces kudriavzevii* (NR_111355.1), *Wickerhamomyces anomalus* (KY105894.1), *Pichia membranifaciens* (NR_111195.1), *Dekkera bruxellensis* (KY103308.1), *Dekkera custersianus* (NR_138184.1), *Yarrowia lipolytica* (KY105973.1), *Kazachstania bulderi* (NR_138198.1), *Kazachstania unispora* (KY103682.1), *Ogataea* sp. (KY104429.1) *Debaryomyces hansenii* (KY103230.1), and *Candida* sp.(NR_137645.1).

Two lambic beer production processes, carried out in two identical oak casks (biological duplicates), were studied as a function of time. The pH, temperature, and apparent attenuation of the wort before its transfer into the casks was 4.3, 17.1°C, and 12.6°P, respectively. Afterward, the pH, temperature, and apparent attenuation profiles of the fermenting wort and maturing beer in both casks were comparable (Figure 1). The pH decreased during the first 2 weeks of fermentation from 4.3 to 3.8, corresponding with enterobacterial and AAB growth, after which a slight increase was noticeable up to pH 4.0 until month 3, suggesting organic acid consumption by yeast species, before it dropped again from month 3 onward to values below pH 3.5 at month 9, corresponding with AAB and LAB acidification (Figures 2–4). The temperature profiles followed the ambient temperature in the cellar and actually reflected the successive seasons. The apparent attenuation of the fermenting wort decreased rapidly in both casks during the first 7 weeks of fermentation, corresponding with ethanol production by yeasts during the main fermentation phase. It remained stable from week 7 until month 6, indicating very low carbohydrate usage, after which a further decrease was noticeable from month 6 onward, corresponding with the maturation phase.

### Microbial Community Dynamics and Species Diversity

The starting wort was spontaneously inoculated overnight during the coolship step and by the brewery equipment used, as indicated by the colony counts on the corresponding mMRS, YPG, and YPGc agar media. They represented presumptive LAB [log 3.0 CFU/ml, including enterobacteria (see below)], yeasts (log 4.0 CFU/ml), and cycloheximide-resistant yeasts (log 3.5 CFU/ml), respectively (Figure 2). AAB were not present, as measured on mDMS agar medium (De Roos et al., 2018).

Based on the counts on these media as a function of time, the lambic beer production processes in both casks studied consisted of four distinctive phases (Figures 2–4). The first phase displayed growth of enterobacteria (first week of the production process), followed by a main fermentation phase (after 24 h till 7 weeks), an acidification phase (week 7 till month 9), and a maturation phase (from month 6 onward). Throughout these phases, a total of 391 bacterial and 1734 yeast isolates were obtained from mMRS agar medium (presumptive LAB) and YPG/YPGc agar media (presumptive yeasts/ cycloheximide-resistant yeasts), respectively, corresponding with 71 plated samples derived from the two lambic beer production casks examined (Figures 3, 4). Only small differences occurred between casks 1 and 2 regarding the microbial community dynamics and species diversity. No differences were found regarding the microbial species diversity for the different sampling heights (top, middle, and bottom of the fermenting wort and maturing beer in the casks).

**FIGURE 5:**
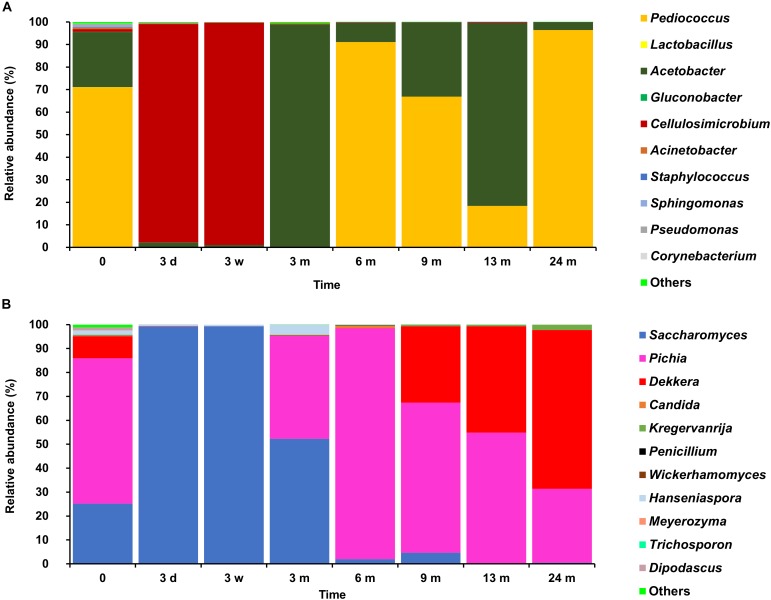
Relative abundances of bacterial **(A)** and fungal **(B)** operational taxonomic units obtained by amplicon sequencing of fermenting wort and maturing beer samples taken from the middle of the cask of the 24-month lambic beer production process conducted in cask 1.

*Klebsiella oxytoca* and *Klebsiella variicola* were most prevalent during the short enterobacterial phase (log 5.0 CFU/ml). These bacteria were isolated from mMRS agar media, indicating that mMRS was not specific enough to isolate LAB solely. During this first phase, a first peak of AAB was found (log 3.5 CFU/ml), whereby *Acetobacter orientalis* was most prevalent (Figures 2–4). High counts of both cycloheximide-resistant yeasts (up to almost log 6.0 CFU/ml), among which *Hanseniaspora uvarum* was most prevalent, and cycloheximide-sensitive yeasts (up to almost log 7.0 CFU/ml), among which *S. cerevisiae* was most prevalent, were found during the initial stages of the main fermentation phase (Figures 2–4). During the first days of fermentation, both *H. uvarum* and *S. cerevisiae* were present in nearly equal counts (up to log 6.0 CFU/ml). Upon further progress of the fermentation, the number of cycloheximide-resistant yeasts decreased rapidly (to log 4.0 CFU/ml), and their species diversity shifted from *H. uvarum* to *Kazachstania* species. The number of cycloheximide-sensitive yeasts reached a maximum after 3 days of fermentation and remained stable for 3 weeks (log 7.0 CFU/ml). Culture-independently, through amplicon sequencing of samples taken from the fermenting wort at the middle of cask 1, mainly *Saccharomyces* and *Cellulosimicrobium* species were found during the first month of fermentation (Figure 5). The occurrence of *Cellulosimicrobium* overlapped with the enterobacterial phase and lasted 2 weeks longer. During the final weeks of the main fermentation phase, the most prevailing yeast species shifted from *S. cerevisiae* to *Saccharomyces kudriavzevii*.

When the number of yeasts decreased, an increase in AAB was found (up to log 6.5 CFU/ml), among which *Acetobacter pasteurianus* was most prevalent (Figures 2–4), and this marked the start of the acidification phase. From month 6 (cask 1) and 7.5 (cask 2) onward, LAB could be isolated, giving counts up to almost log 6.0 CFU/ml (Figure 2). All LAB isolated from mMRS agar media were identified as *P. damnosus*, except at month 24 in cask 1 wherein *Lactobacillus buchneri* was found at low counts (log 3.0 CFU/ml; Figure 3). These culture-dependent data were in agreement with those obtained from the culture-independent analysis performed on samples taken from the fermenting wort at the middle of cask 1, for which LAB were retrieved from month 6 onward (Figure 5). Together with the isolation of high numbers of LAB, also yeast species started to increase again (up to log 6.0 CFU/ml; Figure 2), and pellicle formation became visible.

From month 9 onward, the number of LAB decreased (below log 2.0 CFU/ml) and oxidative yeasts such as *Dekkera* species and *Pichia membranifaciens* co-occurred, as shown by the culture-dependent analysis of casks 1 and 2 (Figures 3, 4) and the culture-independent analysis of cask 1 (Figure 5). *Dekkera* species could be isolated from both YPG and YPGc agar media from months 6 and 9 onward, in casks 1 and 2, respectively, giving counts of up to almost log 6.0 CFU/ml (Figures 2–4). This indicated the start of the maturation phase. From month 18 onward, *Dekkera* species shifted from *D. bruxellensis* to *Dekkera custersianus*, possibly reflecting a higher stress tolerance from the latter yeast species toward the environmental conditions. In cask 1, however, *P. membranifaciens* could be isolated from month 6 onward, although small numbers were probably present as early as month 3, given its detection by culture-independent analysis of the maturing beer in cask 1 from that time point on. In cask 2, *P. membranifaciens* was only isolated from month 9 onward. Opportunistic yeast contaminants such as *Ogataea* species were occasionally detected as well.

### Substrate Consumption and Metabolite Production

Concentrations and profiles of substrates and metabolites were comparable for the biological duplicates analyzed, unless stated otherwise. Since no differences were found regarding substrate and metabolite concentrations for the different sampling heights, concentrations measured in the fermenting wort and maturing beer at the top, middle, and bottom of the casks were averaged.

**FIGURE 6:**
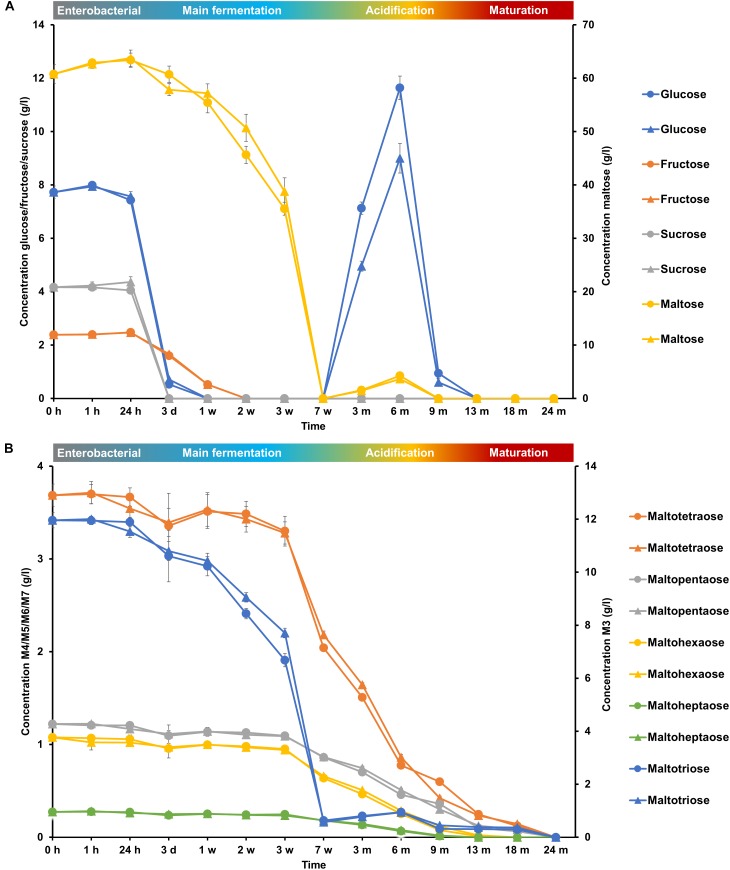
Consumption of **(A)** glucose, fructose, sucrose, and maltose and **(B)** maltotriose (M3), maltotetraose (M4), maltopentaose (M5), maltohexaose (M6), and maltoheptaose (M7) during the 24-month lambic beer production processes carried out in two identical oak casks. Since no differences were found regarding substrates and metabolites for the different sampling heights (top, middle, and bottom of the fermenting wort and maturing beer in the casks), average concentrations are reported. In the case of glucose, fructose, sucrose, maltose, and maltotriose, the averages were taken of the concentrations in the fermenting wort and maturing beer measured at the top, middle, and bottom of the same casks during the same lambic beer production processes reported before (De Roos et al., 2018). Standard deviations represent technical replicates. Cask 1 (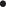) and cask 2 (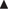).

**FIGURE 7:**
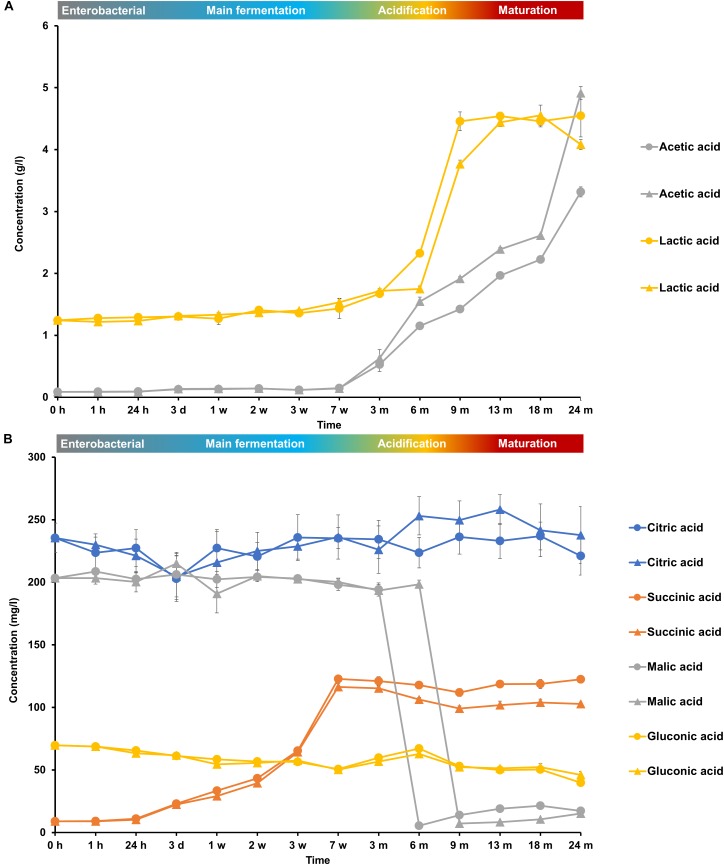
Consumption and production of **(A)** acetic acid and lactic acid and **(B)** citric acid, succinic acid, malic acid, and gluconic acid during the 24-month lambic beer production processes carried out in two identical oak casks. Since no differences were found regarding substrates and metabolites for the different sampling heights (top, middle, and bottom of the fermenting wort and maturing beer in the casks), average concentrations are reported. In the case of acetic acid and gluconic acid, the averages were taken of the concentrations measured in the fermenting wort and maturing beer at the top, middle, and bottom of the same casks during the same lambic beer production processes reported before (De Roos et al., 2018). Standard deviations represent technical replicates. Cask 1 (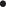) and cask 2 (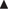).

The initial wort was rich in carbohydrates, among which glucose (8.0 g/l), fructose (2.5 g/l), sucrose (4.0 g/l), maltose (60.0 g/l), maltotriose (12.0 g/l), and higher maltooligosaccharides (<4.0 g/l), were the most abundant (Figure 6). Organic acids, among which lactic acid (1.2 g/l), citric acid (250 mg/l), malic acid (200 mg/l), and gluconic acid (70 mg/l), were also present (Figure 7).

During the enterobacterial phase, no short-chain or branched-chain fatty acids were found, indicating limited growth of the background microbiota. During the main fermentation phase (first 7 weeks), mono-, di-, and trisaccharides were nearly completely depleted. The mono- and disaccharides were consumed sequentially (Figure 6). Sucrose was depleted after 3 days of fermentation, followed by the depletion of glucose and fructose after 1 and 2 weeks, respectively, which corresponded with the enterobacterial and early main fermentation phases. Maltose showed a gradual degradation until it was completely depleted after week 7, coinciding with the alcoholic fermentation phase. Maltotriose followed the same degradation profile as maltose, indicating the ability of both maltose and maltotriose uptake and consumption by the strains of the *Saccharomyces* species present. The exhaustion of both maltose and maltotriose coincided with a decrease in *Saccharomyces* species counts. The higher maltooligosaccharides (from maltotetraose to maltoheptaose) were degraded simultaneously over time, starting from week 3 (Figure 6). Due to the continuous degradation of maltooligosaccharides, combined with the lack of consumption of mono-, di-, and trisaccharides, the concentrations of glucose, maltose, and maltotriose increased from week 7 until month 6, corresponding with the acidification phase (Figure 6).

**FIGURE 8:**
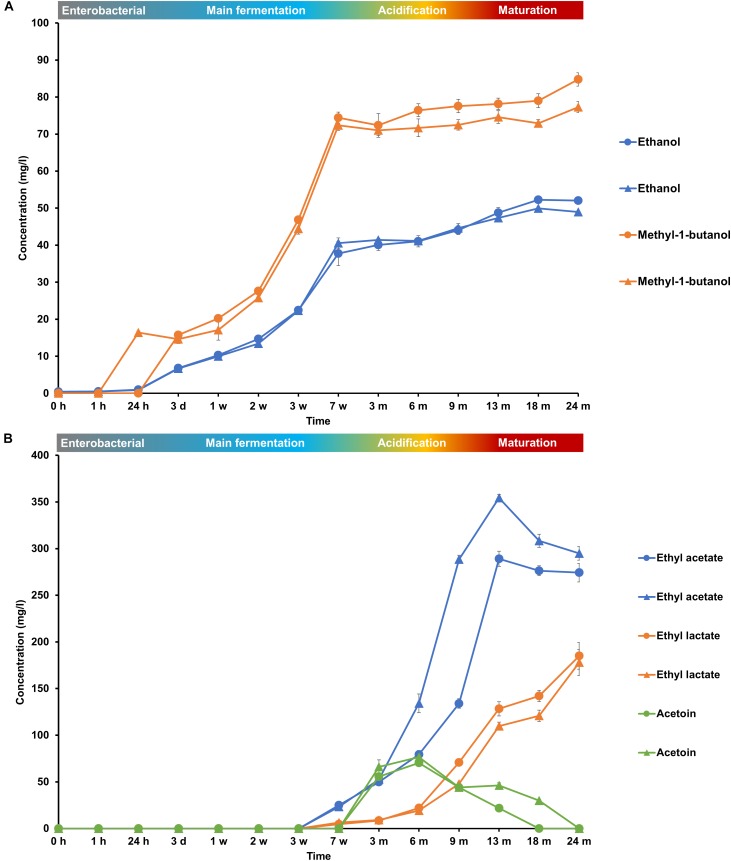
Production of **(A)** ethanol and methyl-1-butanol and **(B)** ethyl acetate, ethyl lactate, and acetoin during the 24-month lambic beer production processes carried out in two identical oak casks. Since no differences were found regarding substrates and metabolites for the different sampling heights (top, middle, and bottom of the fermenting wort and maturing beer in the casks), average concentrations are reported. In the case of ethanol, ethyl acetate and acetoin, the averages were taken of the concentrations measured in the fermenting wort and maturing beer at the top, middle, and bottom of the same casks during the same lambic beer production processes reported before (De Roos et al., 2018). Standard deviations represent technical replicates. Cask 1 (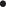) and cask 2 (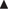).

During the main fermentation phase (first 7 weeks), mostly yeast-associated metabolites such as ethanol, methyl-1-butanol, and succinic acid were produced (Figure 8). From week 3 onward, the most abundant esters of lambic beer, namely ethyl lactate and ethyl acetate, were produced until maximal concentrations of over 100 mg/l and around 300 mg/l, respectively. Isoamyl acetate was not found. Acetoin was produced from 7 weeks until 6 months of fermentation, corresponding with AAB growth, reaching concentrations between 70 and 80 mg/l. Together with the decline of AAB counts, acetoin concentrations decreased after 6 months, indicating consumption by the yeast species present during the maturation phase. 2,3-Butanediol and 2,3-butanedione were not found during the entire production process, indicating no further and full conversion of acetoin, respectively. Acetic acid was produced from week 7 onward, coinciding with the acidification and maturation phases, exceeding concentrations of 2.0 g/l (Figure 7). From month 3 and 6 onward, lactic acid was produced in the fermenting wort of casks 1 and 2, respectively. Both D- and L-lactic acid were produced in nearly equal concentrations (2.0 g/l). Coinciding with the production of lactic acid, malic acid was rapidly depleted after the occurrence of LAB at months 6 and 9 in casks 1 and 2, respectively. After being nearly completely depleted, the concentrations of malic acid slightly increased during the maturation phase, indicating limited malic acid production by the yeast species present. In contrast, citric acid was not metabolized. Gluconic acid was present from the start of the fermentation and a slight increase was noticeable from week 7 until month 6, coinciding with the presence of AAB.

**FIGURE 9:**
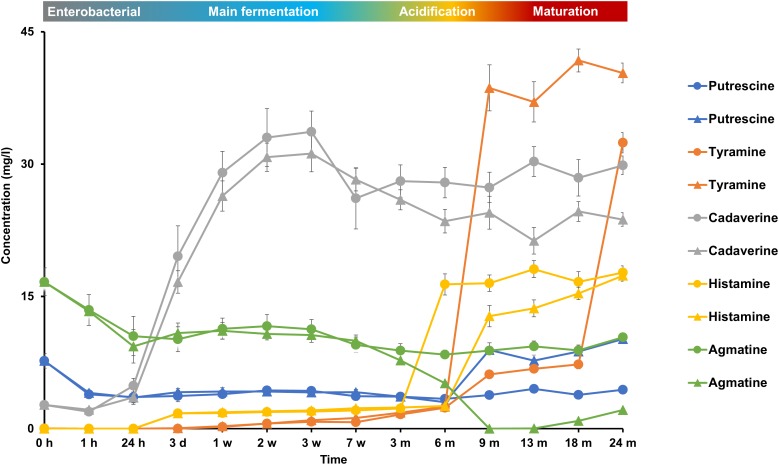
Production and consumption of the biogenic amines putrescine, tyramine, cadaverine, histamine, and agmatine during the 24-month lambic beer production processes carried out in two identical oak casks. Since no differences were found regarding substrates and metabolites for the different sampling heights (top, middle, and bottom of the fermenting wort and maturing beer in the casks), average concentrations are reported. Standard deviations represent technical replicates. Cask 1 (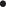) and cask 2 (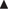).

The initial wort contained some biogenic amines at low concentrations, such as agmatine (9 mg/l), putrescine (8 mg/l), and cadaverine (3 mg/l) (Figure 9). Cadaverine mainly increased during the first week of fermentation, reaching concentrations of over 30 mg/l, indicating its production by the enterobacteria present. Agmatine was completely depleted after month 9 of fermentation in cask 2, whereas it was only partly consumed in cask 1 during the first days of fermentation mainly. Concentration changes of putrescine were negligible throughout the lambic beer production processes. Histamine was produced mainly during the acidification phase after months 3 and 6 in casks 1 and 2, respectively, indicating its production by the LAB species present. Histamine reached concentrations of 18 and 17 mg/l in casks 1 and 2, respectively. Tyramine was the most prevalent biogenic amine present in both casks at the end of the maturation phase and was mainly produced after months 6 and 18 in casks 1 and 2, respectively, reaching concentrations of 34 mg/l and 42 mg/l, respectively. 2-Phenylethylamine and tryptamine were never found.

## Discussion

Traditional lambic beer production processes are characterized by a microbial succession of four groups of microorganisms (Verachtert and Iserentant, 1995; Verachtert and Derdelinckx, 2005; Spitaels et al., 2014; De Roos et al., 2018). Recently performed studies mainly dealt with the microbiology of lambic beer production processes performed in a traditional and common brewery, leading to new insights into this complex spontaneous fermentation and maturation process, but did not tackle physicochemical and metabolomic parameters (Spitaels et al., 2014, 2015). Only the impact of the growth of and substrate consumption and metabolite production by AAB has been underlined recently (De Roos et al., 2018). Therefore, the present study unraveled the dynamics, species diversity, and substrate consumption and metabolite production kinetics of all microbial groups involved in the four consecutive phases of a Belgian, traditional lambic beer production process carried out for 24 months. Moreover, although latest studies have shown that no enterobacterial phase occurs when the wort is acidified (Spitaels et al., 2015), the four distinctive phases of a lambic beer production process were retrieved during the production processes of the present study, albeit that the enterobacterial phase was shorter, spanning the first week of fermentation only, due to the application of manual wort acidification (with lactic acid) before the coolship step. Whether the extent of acidification (depending on the type of acidulant, concentration applied, etc.) or air inoculation (environmental temperature, wort cooling speed, etc.) was responsible for this short enterobacterial phase is not clear. Although the acidification and maturation phases overlapped during the lambic beer production processes of the present study, both phases could be distinguished based on the prevailing growth of AAB and LAB (both causing acidification) versus *Pichia* and *Dekkera* yeasts (marking the start of the maturation phase). It is hypothesized that the extent of separation of these two phases, as well as the distinction between AAB and LAB growth, depends on both the oxygen level and the ambient temperature. The oxygen level is of importance to promote AAB growth (Pothakos et al., 2016; De Roos et al., 2018). LAB need a temperature above 15°C to be able to grow during lambic beer production (De Keersmaecker, 1996). Except for AAB growth and the production of AAB-related metabolites that took place mainly at the liquid/air interphase of the casks, no spatial differences occurred regarding yeast and LAB species diversity, substrate consumption, and metabolite production in the fermenting wort and maturing beer. As inoculation of the wort occurred before it was transferred into the casks, the microorganisms probably originated from the brewery air during cooling, contact with transfer tubings, or a combination of both. However, since previous research only recovered few yeasts or bacteria relevant for lambic beer fermentation from the coolship or brewery air, it was more likely that a meaningful inoculation occurred when the wort came into contact with other brewery surfaces (Bokulich et al., 2015; Spitaels et al., 2015). Tubings used for both matured lambic beer and fresh wort and other non-production equipment that encountered wort/matured lambic beer are often cleaned superficially and may thus harbor fermentation-related microorganisms. It has indeed been shown that substrate contact is the main actor in shaping the microbial community structures of different brewery surfaces (Bokulich et al., 2015). Recently, it has been shown that the wooden barrels can be an additional inoculation source too (De Roos et al., 2019).

During the lambic beer production processes studied, the prevalence of specific microbial species shifted as a function of time, which depended on a combination of both substrate consumption and metabolite production as well as environmental factors such as temperature and pH. The enterobacteria present from the start of the fermentation disappeared quickly, due to increasing ethanol concentrations produced by the yeasts, decreasing pH values because of enterobacterial and AAB (*A. orientalis*) acidification, and monosaccharide depletion by microbial growth in general (Priest and Stewart, 2006; De Roos et al., 2018). Besides enterobacteria and AAB, *Cellulosimicrobium* species occurred during the enterobacterial phase (shown culture-independently only). However, since *Cellulosimicrobium* was never encountered before in lambic beer fermentations and yeast species generally dominate the main fermentation phase, it is likely that the bacterial DNA concentrations of these samples are very low and that the enzymes lyticase and Zymolyase used in the DNA extraction protocol may be the main source for these sequencing reads. Lyticase and Zymolyase are typically isolated from *Cellulosimicrobium cellulans* and this species has been found upon DNA extraction and high-throughput sequencing frequently (Ferrer, 2006; Verce et al., unpublished results).

Both *H. uvarum* and *S. cerevisiae* initiated the main fermentation phase. Fast consumption of the carbohydrate substrates available and the production of high ethanol concentrations led *S. cerevisiae* to take over from other yeast species, as this impaired growth of competing microorganisms (De Keersmaecker, 1996; Piškur et al., 2006; Rozpedowska et al., 2011; Spitaels et al., 2015). The transition from *H. uvarum* to *S. cerevisiae* is well described during spontaneous wine fermentations and is most likely caused by the production of certain *S. cerevisiae* metabolites, such as inhibitory ethanol and antimicrobial peptides (Branco et al., 2014; Wang et al., 2015). The slower decrease of other cycloheximide-resistant yeasts, such as *Kazachstania* species, indicated that such inhibitory effects are less pronounced, when compared with the Crabtree-negative *H. uvarum* (Hagman and Piškur, 2015). The occurrence of *H. uvarum* during the initial stages of the lambic beer fermentation process has been described before (Van Oevelen et al., 1977; Verachtert and Iserentant, 1995). In contrast, lambic beer fermentations do occur in the absence of *H. uvarum* (Spitaels et al., 2014, 2015). Hence, *H. uvarum* may not be necessary for lambic beer production, although it is known that *S. cerevisiae* is metabolically stimulated, under aerobic and anaerobic conditions, in co-cultivation with non-*Saccharomyces* yeasts (Curiel et al., 2017). Furthermore, *Saccharomyces* species are indispensable and are known to dominate the early stages of lambic beer fermentation, due to their fast carbohydrate consumption, ethanol production, accumulation and tolerance, and ability to ferment in the absence of oxygen (De Keersmaecker, 1996; Piškur et al., 2006; Spitaels et al., 2015). Probably due to an environmental temperature drop of the fermenting wort, the yeast species diversity shifted from the mesophilic *S. cerevisiae* to the cryotolerant *S. kudriavzevii*. The presence of *S. kudriavzevii* was not surprising, as this yeast species is associated with oak bark and can co-exist with *S. cerevisiae* and *Saccharomyces paradoxus* (Sampaioa and Goncalves, 2008). Moreover, *S. cerevisiae-S. kudriavzevii* hybrids are common in Belgian-style beers (González et al., 2008).

The main fermentation phase was characterized by a sequential consumption of mono-, di-, and trisaccharides and high production of yeast-associated metabolites, such as ethanol, succinic acid, and methyl-1-butanol. Exhaustion of maltose and maltotriose caused starvation of *Saccharomyces* species and was followed by the acidification phase. Also simultaneous and continuous breakdown of maltooligosaccharides occurred during this acidification phase. Although *Dekkera* species produce both intra- and extracellular α-glucosidases, enzymes capable of dextrin degradation (Shanta Kumara et al., 1993), these species were only isolated from month 6 onward, whereas the continuous breakdown of maltooligosaccharides already started during the first month of fermentation. Moreover, it has been shown that the degradation of lower maltooligosaccharides happens faster than that of higher maltooligosaccharides when *Dekkera* species are involved (Shanta Kumara et al., 1993). Therefore, the breakdown of maltooligosaccharides during the acidification phase and even earlier was most likely due to the presence of yeast species at low counts (<2.0 log CFU/ml) or as a result of cell lysis of yeast species present during the main fermentation phase, thereby releasing dextrin-degrading enzymes into the fermenting wort. Also acid hydrolysis cannot be ruled out, given the length of the lambic beer production process.

Acidification was partly due to AAB species, among which *A. pasteurianus* was most prevalent. The high production of acetic acid by AAB through oxidation of ethanol produced by the yeasts led to the formation of the ester ethyl acetate, probably due to a combination of enzymatic and chemical reactions (De Roos et al., 2018). Since no 2,3-butanedione or 2,3-butanediol occurred in the absence of LAB and presence of AAB during the first months of the acidification phase, the production of acetoin was due to the oxidation of lactic acid by AAB (Moens et al., 2014; De Roos et al., 2018). The preference of *Acetobacteraceae* to grow on ethanol and the continuous breakdown of maltooligosaccharides led to the elaboration of glucose, maltose, and maltotriose during the first part of the acidification phase, after which they were consumed by the LAB and yeasts. The later stages of the acidification phase allowed the growth of the LAB species *P. damnosus*, thanks to the increased environmental temperature. This led to lactic acid production and the formation of the ester ethyl lactate, which was again probably a combined effect of both chemical and enzymatic reactions. Whereas co-fermentation of glucose and malic acid took place by the LAB species present, citric acid was not metabolized. Indeed, it has been shown that for wine fermentations *Pediococcus parvulus* for example does not degrade citric acid during malolactic fermentation, as opposed to *Oenococcus oeni* (Davis et al., 1986). Although malic acid consumption has been reported in different yeast species as well (Su et al., 2014), it is unlikely to be the case here, since *P. membranifaciens* was the only yeast species already detected in the fermenting wort in both casks before malic acid was consumed. Moreover, during the maturation phase, when *P. membranifaciens* co-occurred with *Dekkera* species, malic acid production rather than its consumption occurred. Up to now, malolactic fermentation was never reported during lambic beer production. Malolactic fermentation generally occurs in spontaneously fermented alcoholic beverages, such as wine (González-Arenzana et al., 2012, 2013a,b) and cider (Salih et al., 1988; Swaffield and Scott, 1995), and is carried out by many LAB species during fermentation of vegetables and fruits (Axelsson, 2004). It is performed in the commercial production of wine to convert tart L-malic acid into softer-tasting L-lactic acid (Fugelsang and Edwards, 2007) and can for the same reason be of importance during lambic beer production.

Biogenic amines were present at very low concentrations in the lambic brews of the present study, thanks to the limited growth of enterobacteria. In contrast, cheese and fermented sausage can contain much higher biogenic amine concentrations (EFSA Panel on Biological Hazards, 2011; Schirone et al., 2012; Latorre-Moratalla et al., 2017). *P. damnosus* also contributed to biogenic amine (histamine) formation, although staying well below the concentrations that are acceptable for health (<400 mg/kg). As is the case in most fermented foods, histamine was present together with tyramine in the lambic beer production processes of the present study (Alvarez and Moreno-Arribas, 2014; del Rio et al., 2017). Although tyramine is known to be produced in beer by some *Pediococcus* species (Izquierdo-Pulido et al., 1997), it only increased after their prevalence during the acidification phase. It is possible that tyramine is produced by other LAB species present that were too low in abundance to be detected culture-dependently or culture-independently. Another possibility is the release of tyramine into the fermenting wort and maturing beer due to autolysis of yeast cells (Blackwell et al., 1969; Bonnin-Jusserand et al., 2012).

Lactic acid bacteria and *Dekkera* species occurred together during the first months of the maturation phase. Co-occurrence of both microbial groups causes a more pronounced over-attenuation (Shanta Kumara and Verachtert, 1991). *Dekkera* species and *P. membranifaciens* grew during the maturation phase, thereby consuming mainly carbohydrates to produce additional ethanol. The yeast species of the maturation phase were further responsible for pellicle formation, in turn decreasing the oxygen influx and therefore causing a decrease in AAB numbers (De Roos et al., 2018). *Dekkera* species are undesirable in wine but indispensable in lambic beer and Orval trappist productions. They have an important effect on the volatile aroma profile of lambic beers with, for example, the formation of ethyl acetate, ethyl lactate and phenethyl acetate, along with the hydrolysis of isoamyl acetate (Spaepen and Verachtert, 1982). They are also capable of producing 4-ethylguaiacol and 4-ethylphenol, known as Brett flavor in lambic beer and Orval, under conditions of little residual carbohydrates during the maturation process (Chatonnet et al., 1995; Lentz and Harris, 2015). During the maturation phase, acetoin concentrations diminished, probably due to the use of acetoin, produced by AAB, as an alternative external electron acceptor by *Dekkera* species to reconcile their redox imbalance caused by their incapability to reoxidize NADH + H^+^ via the glycerol pathway under oxygen limitation (Steensels et al., 2015; De Roos et al., 2018).

## Conclusion

The present study monitored the temporal and spatial distribution of microorganisms, substrates, and metabolites during lambic beer production processes, thereby unraveling reasons behind certain community dynamics such as the transition of *Hanseniaspora* and *Kazachstania* species to *Saccharomyces* species, and the community dynamics of AAB, LAB, and yeasts during the acidification and maturation phases. Moreover, some new fermentation characteristics during lambic beer production were revealed, such as the occurrence of a malolactic fermentation by *P. damnosus*, the consumption of acetoin by *Dekkera* species, the simultaneous breakdown of maltooligosaccharides possibly by the yeasts, and the occurrence of a short enterobacterial phase despite manual wort acidification with lactic acid. In most lambic beer breweries, the latter is applied to enhance the microbial stability of the wort and to control biogenic amine production by enterobacteria. However, the data of the present study showed that, although the enterobacterial phase was shortened by this application, biogenic amines were still produced during the overnight cooling of the wort and the early stages of fermentation, albeit in very low concentrations, which do not have to be worried about. Moreover, also other microbial groups such as LAB contributed to the total biogenic amine contents. Therefore, wort acidification does not avoid biogenic amine production completely, although it contributes to the controllability of the biogenic amine production in particular and of the lambic beer production process in general by enhancing the microbial stability without hampering the fermentation and maturation process. This new knowledge of how the microbial communities are shaped throughout the lambic beer production process will contribute to its follow-up, which is mainly based on human sensory analysis nowadays.

## Author Contributions

JR contributed to the experimental work, the acquisition, processing, and interpretation of the data, and drafting of the manuscript. PV and LV interpreted the data, supervised the work, and reviewed and edited the manuscript.

## Conflict of Interest Statement

The authors declare that the research was conducted in the absence of any commercial or financial relationships that could be construed as a potential conflict of interest.

## Footnotes

1 http://blast.ncbi.nlm.nih.gov/

## References

[B1] Altschul S. F. Gish W. Miller W. Myers E. W. Lipman D. J. (1990). Basic local alignment search tool. *J. Mol. Biol.* 215 403–410. 10.1016/S0022-2836(05)80360-2 2231712

[B2] Alvarez M. A. Moreno-Arribas V. (2014). The problem of biogenic amines in fermented foods and the use of potential biogenic amine-degrading microorganisms as a solution. *Trends Food Sci. Technol.* 39 146–155. 10.1016/j.tifs.2014.07.007

[B3] Axelsson L. (2004). “Lactic acid bacteria: classification and physiology,” in *Lactic Acid Bacteria, Microbiological and Functional Aspects*, eds Salminen S. von Wright A. Ouwehand A. (New York, NY: Marcel Dekker), 1–66. 10.1201/9780824752033.ch1

[B4] Blackwell B. Mabitt L. A. Marley E. (1969). Histamine and tyramine content of yeast products. *J. Food Sci.* 34 47–51. 10.1111/j.1365-2621.1969.tb14359.x

[B5] Bokulich N. A. Bamforth C. W. (2013). The microbiology of malting and brewing. *Microbiol. Mol. Biol. Rev.* 77 157–172. 10.1128/MMBR.00060-12 23699253 PMC3668669

[B6] Bokulich N. A. Bamforth C. W. (2017). *Brewing Microbiology: Current Research, Omics and Microbial Ecology.* Poole: Caister AcademicPress, 332.

[B7] Bokulich N. A. Bamforth C. W. Mills D. A. (2012). Brewhouse-resident microbiota are responsible for multi-stage fermentation of American coolship ale. *PLoS One* 7:e35507. 10.1371/journal.pone.0035507 22530036 PMC3329477

[B8] Bokulich N. A. Bergsveinson J. Ziola B. Mills D. A. (2015). Mapping microbial ecosystems and spoilage-gene flow in breweries highlights patterns of contamination and resistance. *eLife* 4:e04634. 10.7554/eLife.04634 25756611 PMC4352708

[B9] Bokulich N. A. Mills D. A. (2013). Improved selection of internal transcribed spacer-specific primers enables quantitative, ultra-high-throughput profiling of fungal communities. *Appl. Environ. Microbiol.* 79 2519–2526. 10.1128/AEM.03870-12 23377949 PMC3623200

[B10] Bonnin-Jusserand M. Grandvalet C. Rieu A. Weidmann S. Alexandre H. (2012). Tyrosine-containing peptides are precursors of tyramine produced by Lactobacillus plantarum strain IR BL0076 isolated from wine. *BMC Microbiol.* 12 199–210. 10.1186/1471-2180-12-199 22963406 PMC3492074

[B11] Branco P. Francisco D. Chambon C. Hébraud M. Arneborg N. Almeida M. G. (2014). Identification of novel GAPDH-derived antimicrobial peptides secreted by Saccharomyces cerevisiae and involved in wine microbial interactions. *Appl. Microbiol. Biotechnol.* 98 843–853. 10.1007/s00253-013-5411-y 24292082

[B12] Briggs D. E. Boulton C. Brookes P. Stevens R. (2004). *Brewing Science and Practice.* Cambridge: Woodhead Publishing Limited, 900. 10.1201/9780203024195

[B13] Caporaso J. G. Lauber C. L. Walters W. A. Berg-Lyons D. Lozupone C. A. Turnbaugh P. J. (2011). Global patterns of 16S rRNA diversity at a depth of millions of sequences per sample. *Proc. Natl. Acad. Sci. U.S.A.* 108 4516–4522. 10.1073/pnas.1000080107 20534432 PMC3063599

[B14] Chatonnet P. Dubourdieu D. Boidron J. N. (1995). The influence of Brettanomyces/Dekkera sp. yeasts and lactic acid bacteria on the ethylphenol content of red wines. *Am. J. Enol. Viticult.* 46 463–468. 10.1007/s00253-013-5411-y 24292082

[B15] Curiel J. A. Morales P. Gonzalez R. Tronchoni J. (2017). Different non-Saccharomyces yeast species stimulate nutrient consumption in *S. cerevisiae* mixed cultures. *Front. Microbiol.* 8:2121. 10.3389/fmicb.2017.02121 29163412 PMC5671574

[B16] Davis C. R. Wibowo D. J. Lee T. H. Fleet G. H. (1986). Growth and metabolism of lactic acid bacteria during and after malolactic fermentation of wines at different pH. *Appl. Environ. Microbiol.* 51 539–545. 16347015 10.1128/aem.51.3.539-545.1986 PMC238915

[B17] De Bruyn F. Zhang S. Pothakos V. Torres J. Lambot C. Moroni A. V. (2017). Exploring the impact of post-harvest processing on the microbiota and metabolite profiles during a case of green coffee bean production. *Appl. Environ. Microbiol.* 83:e002398-16. 10.1128/AEM.02398-16 PMC5165123 27793826

[B18] De Keersmaecker J. (1996). The mystery of lambic beer. *Sci. Am.* 275 74–81. 10.1038/scientificamerican0896-74

[B19] De Roos J. De Vuyst L. (2018). Acetic acid bacteria in fermented foods and beverages. *Curr. Opin. Biotechnol.* 49 115–119. 10.1016/j.copbio.2017.08.007 28863341

[B20] De Roos J. Van der Veken D. De Vuyst L. (2019). The interior surfaces of wooden barrels are an additional microbial inoculation source for lambic beer production. *Appl. Environ. Microbiol.* 85:e02226-18. 10.1128/AEM.02226-18 30389768 PMC6293109

[B21] De Roos J. Verce M. Aerts M. Vandamme P. De Vuyst L. (2018). Temporal and spatial distribution of the acetic acid bacterium communities throughout the wooden casks used for the fermentation and maturation of lambic beer underlines their functional role. *Appl. Environ. Microbiol.* 84:e02846-17. 10.1128/AEM.02846-17 29352086 PMC5861831

[B22] del Rio B. Redruello B. Linares D. M. Ladero V. Fernandez M. Martin M. C. (2017). The dietary biogenic amines tyramine and histamine show synergistic toxicity towards intestinal cells in culture. *Food Chem.* 218 249–255. 10.1016/j.foodchem.2016.09.046 27719906

[B23] Edgar R. C. Haas B. J. Clemente J. C. Quince C. Knight R. (2011). UCHIME improves sensitivity and speed of chimera detection. *Bioinformatics* 27 2194–2200. 10.1093/bioinformatics/btr381 21700674 PMC3150044

[B24] EFSA Panel on Biological Hazards. (2011). Scientific opinion on scientific opinion on risk based control of biogenic amine formation in fermented foods. *EFSA J.* 9:2393 10.2903/j.efsa.2011.2393

[B25] Ferrer P. (2006). Revisiting the *Cellulosimicrobium cellulans* yeast-lytic β-1,3-glucanases toolbox: a review. *Microb. Cell Fact.* 5:10. 10.1186/1475-2859-5-10 16545129 PMC1458353

[B26] Findley K. Oh J. Yang J. Conlan S. Deming C. Meyer J. A. (2013). Topographic diversity of fungal and bacterial communities in human skin. *Nature* 498 367–370. 10.1038/nature12171 23698366 PMC3711185

[B27] Fugelsang K. C. Edwards C. G. (2007). “Microbial ecology during vinification,” in *Wine Microbiology*, eds Fugelsang K. C. Edwards C. G. (New York, NY: Springer), 82–101.

[B28] González-Arenzana L. López R. Santamaría P. Tenorio C. López-Alfaro I. (2012). Dynamics of indigenous lactic acid bacteria populations in wine fermentations from La Rioja (Spain) during three vintages. *Microb. Ecol.* 63 12–19. 10.1007/s00248-011-9911-y 21779812

[B29] González S. S. Bario E. Querol A. (2008). Molecular characterization of new natural hybrids of *Saccharomyces cerevisiae* and *S. kudriavzevii* in brewing. *Appl. Microbiol. Biotechnol.* 74 2314–2320. 10.1128/AEM.01867-07 18296532 PMC2293171

[B30] González-Arenzana L. López R. Santamaría P. López-Alfaro I. (2013a). Dynamics of lactic acid bacteria populations in Rioja wines by PCR-DGGE, comparison with culture-dependent methods. *Appl. Microbiol. Biotechnol.* 97 6931–6941. 10.1007/s00253-013-4974-y 23685477

[B31] González-Arenzana L. Santamaría P. López R. López-Alfaro I. (2013b). Indigenous lactic acid bacteria communities in alcoholic and malolactic fermentations of Tempranillo wines elaborated in ten wineries of La Rioja (Spain). *Food Res. Int.* 50 438–445. 10.1016/j.foodres.2012.11.008

[B32] Gweon H. S. Oliver A. Taylor J. Booth T. Gibbs M. Read D. S. (2015). PIPITS: an automated pipeline for analyses of fungal internal transcribed spacer sequences from the Illumina sequencing platform. *Methods Ecol. Evol.* 6 973–980. 10.1111/2041-210X.12399 27570615 PMC4981123

[B33] Hagman A. Piškur J. (2015). A study on the fundamental mechanism and the evolutionary driving forces behind aerobic fermentation in yeast. *PLoS One* 10:e0116942. 10.1371/journal.pone.0116942 25617754 PMC4305316

[B34] Howard P. H. (2014). “Too big to ale? Globalization and consolidation in the beer industry,” in *The Geography of Beer: Regions, Environment, and Society*, eds Patterson M. Pullen N. (New York, NY: Springer), 155–165.

[B35] Izquierdo-Pulido M. Carceller-Rosa J.-M. Maine-Font A. Vidal-Caroue M. C. (1997). Tyramine formation by *Pediococcus* spp. during beer fermentation. *J. Food Protect.* 60 831–836. 10.4315/0362-028X-60.7.831 31026895

[B36] Izquierdo-Pulido M. Font-Fábregas J. Vidal-Carou C. (1995). Influence of *Saccharomyces cerevisiae* var. uvarum on histamine and tyramine formation during beer fermentation. *Food Chem.* 54 51–54. 10.1016/0308-8146(95)92661-3

[B37] Izquierdo-Pulido M. Hernández-Jover T. Mariné-Font A. Vidal-Carou M. C. (1996). Biogenic amines in European beers. *J. Agric. Food Chem.* 44 3159–3163. 10.1021/jf960155j 14561522

[B38] Kalac P. Krizek M. (2003). A review of biogenic amines and polyamines in beer. *J. Instit. Brew.* 109 123–128. 10.1002/j.2050-0416.2003.tb00141.x

[B39] Latorre-Moratalla M. L. Comas-Basté O. Bover-Cid S. Vidal-Carou M. C. (2017). Tyramine and histamine risk assessment related to consumption of dry fermented sausages by the Spanish population. *Food Chem. Toxicol.* 99 78–85. 10.1016/j.fct.2016.11.011 27856296

[B40] Laureys D. De Vuyst L. (2014). Microbial species diversity, community dynamics, and metabolite kinetics of water kefir fermentation. *Appl. Environ. Microbiol.* 80 2564–2572. 10.1128/AEM.03978-13 24532061 PMC3993195

[B41] Lentz M. Harris C. (2015). Analysis of growth inhibition and metabolism of hydroxycinnamic acids by brewing and spoilage strains of Brettanomyces yeast. *Foods* 4 581–593. 10.3390/foods4040581 28231223 PMC5224551

[B42] Martens H. Dawoud E. Verachtert H. (1991). Wort enterobacteria and other microbial populations involved during the 1st month of lambic fermentation. *J. Instit. Brew.* 97 435–439. 10.1002/j.2050-0416.1991.tb01082.x

[B43] Martens H. Dawoud E. Verachtert H. (1992). Synthesis of aroma compounds by wort enterobacteria during the 1st stage of lambic fermentation. *J. Instit. Brew.* 98 421–425. 10.1002/j.2050-0416.1992.tb01126.x

[B44] Martens H. Iserentant D. Verachtert H. (1997). Microbiological aspects of a mixed yeast-bacterial fermentation in the production of a special Belgian acidic ale. *J. Instit. Brew.* 103 85–91. 10.1002/j.2050-0416.1997.tb00939.x

[B45] Martin M. (2011). Cutadapt removes adapter sequences from high-throughput sequencing reads. *EMBnet J.* 17 10–12. 10.14806/ej.17.1.200

[B46] Moens F. Lefeber T. De Vuyst L. (2014). Oxidation of metabolites highlights the microbial interactions and role of *Acetobacter pasteurianus* during cocoa bean fermentation. *Appl. Environ. Microbiol.* 80 1848–1857. 10.1128/AEM.03344-13 24413595 PMC3957632

[B47] Oelofse A. Pretorius I. S. du Toit M. (2008). Significance of Brettanomyces and Dekkera during winemaking: a synoptic review. *South Afr. J. Enol. Viticult.* 29 128–144.

[B48] Piškur J. Rozpȩdowska E. Polakova S. Merico A. Compagno C. (2006). How did Saccharomyces evolve to become a good brewer? *Trends Genet.* 22 183–186. 10.1016/j.tig.2006.02.002 16499989

[B49] Pothakos V. Illeghems K. Laureys D. Spitaels F. Vandamme P. De Vuyst L. (2016). “*Acetic acid bacteria in fermented food and beverage ecosystems*,” in *Acetic Acid Bacteria*: Ecology and Physiology, eds Matsushita K. Toyama H. Tonouchi N. Okamoto-Kainuma A. (Tokyo: Springer), 73–100.

[B50] Priest F. G. Stewart G. G. (2006). “Microbiology and microbiological control in the brewery,” in *Handbook of Brewing*, 2nd Edn, eds Priest F. G. Stewart G. G. (Boca Raton, FL: CRC Press), 607–629.

[B51] Rozpedowska E. Hellborg L. Ishchuk O. P. Orhan F. Galafassi S. Merico A. (2011). Parallel evolution of the make-accumulate-consume strategy in Saccharomyces and Dekkera yeasts. *Nat. Commun.* 2:302. 10.1038/ncomms1305 21556056 PMC3112538

[B52] Salih A. Drilleau J. Cavin F. Divies C. Bourgeois C. (1988). A survey of microbiological aspects of cider making. *J. Instit. Brew.* 94 5–8. 10.1002/j.2050-0416.1988.tb04545.x

[B53] Sampaioa J. P. Goncalves P. (2008). Natural populations of *Saccharomyces kudriavzevii* in Portugal are associated with oak bark and are sympatric with *S. cerevisiae* and *S. paradoxus*. *Appl. Environ. Microbiol.* 77 2144–2152. 10.1128/AEM.02396-07 18281431 PMC2292605

[B54] Schirone M. Tofalo R. Visciano P. Corsetti A. Suzzi G. (2012). Biogenic amines in Italian Pecorino cheese. *Front. Microbiol.* 3:171 10.3389/fmicb.2012.00171 PMC3347038 22586425

[B55] Schloss P. D. Westcott S. L. Ryabin T. Hall J. R. Hartmann M. Hollister E. B. (2009). Introducing Mothur: open-source, platform-independent, community-supported software for describing and comparing microbial communities. *Appl. Environ. Microbiol.* 75 7537–7541. 10.1128/AEM.01541-09 19801464 PMC2786419

[B56] Shanta Kumara H. M. C. Decort S. Verachtert H. (1993). Localization and characterization of α-glucosidase activity in *Brettanomyces lambicus*. *Appl. Environ. Microbiol.* 59 2352–2358. 16349005 10.1128/aem.59.8.2352-2358.1993 PMC182290

[B57] Shanta Kumara H. M. C. Verachtert H. (1991). Identification of lambic superattenuating microorganisms by the use of selective antibiotics. *J. Instit. Brew.* 97 181–185. 10.1002/j.2050-0416.1991.tb01064.x

[B58] Snauwaert I. Roels S. P. Van Nieuwerburg F. Van Landschoot A. De Vuyst L. Vandamme P. (2016). Microbial diversity and metabolite composition of Belgian red-brown acidic ales. *Int. J. Food Microbiol.* 221 1–11. 10.1016/j.ijfoodmicro.2015.12.009 26802571

[B59] Spaepen M. Van Oevelen D. Verachtert H. (1978). Fatty acids and esters produced during the spontaneous fermentation of lambic and gueuze. *J. Instit. Brew.* 84 278–282. 10.1002/j.2050-0416.1978.tb03888.x

[B60] Spaepen M. Van Oevelen D. Verachtert H. (1979). Higher fatty acid (HFA) and HFA-ester content of spontaneously fermented Belgian beers and evaluation of their analytical determination. *Brauwissenschaft* 32 S1–S6.

[B61] Spaepen M. Verachtert H. (1982). Esterase activity in the genus Brettanomyces. *J. Instit. Brew.* 88 11–17. 10.1002/j.2050-0416.1982.tb04061.x

[B62] Spano G. Russo P. Lonvaud-Funel A. Lucas P. Alexandre H. Grandvalet C. (2010). Biogenic amines in fermented foods. *Eur. J. Clin. Nutr.* 64 S95–S100. 10.1038/ejcn.2010.218 21045859

[B63] Spitaels F. Wieme A. D. Janssens M. Aerts M. Daniel H.-M. Van Landschoot A. (2014). The microbial diversity of traditional spontaneously fermented lambic beer. *PLoS One* 9:e95384. 10.1371/journal.pone.0095384 24748344 PMC3991685

[B64] Spitaels F. Wieme A. D. Janssens M. Aerts M. Van Landschoot A. De Vuyst L. (2015). The microbial diversity of an industrially produced lambic beer shares members of a traditionally produced one and reveals a core microbiota for lambic beer fermentation. *Food Microbiol.* 49 23–32. 10.1016/j.fm.2015.01.008 25846912

[B65] Spitaels F. Wieme A. D. Snauwaert I. De Vuyst L. Vandamme P. (2017). “Microbial ecology of traditional beer fermentations,” in *Brewing Microbiology: Current Research, Omics and Microbial Ecology*, eds Bokulich N. Bamforth C. (Poole: Caister Academic Press), 179–196.

[B66] Steensels J. Daenen L. Malcorps P. Derdelinckx G. Verachtert H. Verstrepen K. J. (2015). Brettanomyces yeasts – From spoilage organisms to valuable contributors to industrial fermentations. *Int. J. Food Microbiol.* 206 24–38. 10.1016/j.ijfoodmicro.2015.04.005 25916511

[B67] Su J. Wang T. Wang Y. Li Y.-Y. Li H. (2014). The use of lactic acid-producing, malic acid-producing, or malic acid-degrading yeast strains for acidity adjustment in the wine industry. *Appl. Microbiol. Biotechnol.* 98 2395–2413. 10.1007/s00253-014-5508-y 24430209

[B68] Swaffield C. H. Scott J. A. (1995). Existence and development of natural microbial populations in wooden storage vats used for alcoholic cider maturation. *J. Am. Soc. Brew. Chem.* 53 117–120. 10.1094/ASBCJ-53-0117

[B69] Van Oevelen D. L’Escaille F. Verachtert H. (1976). Synthesis of aroma components during the spontaneous fermentation of lambic and gueuze. *J. Instit. Brew.* 82 322–326. 10.1002/j.2050-0416.1975.tb06953.x

[B70] Van Oevelen D. Spaepen M. Timmermans P. Verachtert H. (1977). Microbiological aspects of spontaneous wort fermentation in the production of lambic and gueuze. . *J. Instit. Brew.* 83 356–360. 10.1002/j.2050-0416.1977.tb03825.x

[B71] Verachtert H. (1983). De spontane geuzegisting - La fermentation spontanée de la geuze. *Cerevisia Belgian J. Brew. Biotechnol.* 8 41–48.

[B72] Verachtert H. Dawoud E. (1984). Microbiology of lambic-type beers. *J. Appl. Bacteriol.* 57 R11–R12. 7595636

[B73] Verachtert H. Dawoud E. Kumara H. M. C. S. (1989). Interactions between *Enterobacteriaceae* and *Saccharomyces cerevisiae* during wort fermentation. *Yeast* 5 67–72. 19601666

[B74] Verachtert H. Derdelinckx G. (2005). Acidic beers: enjoyable reminiscences of the past. *Cerevisia Belgian J. Brew. Biotechnol.* 30 38–47.

[B75] Verachtert H. Iserentant D. (1995). Properties of Belgian acid beers and their microflora. Part I. The production of gueuze and related refreshing acid beers. *Cerevisia Belgian J. Brew. Biotechnol.* 20 37–41.

[B76] Vermote L. Verce M. De Vuyst L. Weckx S. (2018). Amplicon and shotgun metagenomic sequencing indicates that microbial ecosystems present in cheese brines reflect environmental inoculation during the cheese production process. *Int. Dairy J.* 87 44–53. 10.1016/j.idairyj.2018.07.010

[B77] Wang C. Albert M. Esteve-Zarzoso B. (2015). Interaction between Hanseniaspora uvarum and *Saccharomyces cerevisiae* during alcoholic fermentation. *Int. J. Food Microbiol.* 206 67–74. 10.1016/j.ijfoodmicro.2015.04.022 25956738

